# Medical New York

**Published:** 1896-10

**Authors:** L. B. Grandy

**Affiliations:** New York


### CORRESPONDENCE.

### MEDICAL NEW YORK.

NEW YORK, September 15, 1896.

With the approach of cooler weather there is every evidence of returning activity on every hand—social, commercial, and medical. To-day witnesses the opening of the winter sessions of the Polyclinic and Postgraduate, and each institution begins with a large number of matriculates from the interior, who are seeking to replenish their stores of medical and surgical knowledge. The last sessions of these schools have been well attended and particularly successful. The increasing number of physicians who attend these schools annually amply justifies their existence, showing that they supply a real want which is realized by the great masses of practitioners throughout the country.

Much indignation has been felt and expressed over what has been termed the patent medicine scandal at Bellevue Hospital. The Commissioners of Public Charities set apart a ward in the hospital for the trial of a secret remedy for drunkenness, alleged to have been discovered by a New York physician. The protests of the regular medical staff were absolutely ignored, and the physician (who was not a member of the staff) allowed to experiment with his supposed remedy.

The Medical News was particularly energetic in denouncing such unusual and high-handed procedure on the part of the Commissioners, and it called attention to the important fact that the so-called cure was the property of a stock company, which was seeking to exploit the remedy under the auspices and sanction of the hospital. That this *sub-rosa* investigation was suddenly discontinued after about two months' fruitless trial seems to have been due, not so much to lack of sympathy and encouragement on the part of the Commissioners, as to lack of energy and perseverance on the part of Dr. Oppenheimer, the alleged discoverer and chief promoter. After the death of two or three patients he abandoned the remainder, and the secret cure, which had been so tenderly fostered by the kindness of the Commissioners, was left as a foundling upon the doorstep of the hospital. So at present the relations between the medical board of the hospital and the guileless gentlemen who are the Commissioners of Charities under the reform government are naturally somewhat strained.

Physicians coming to New York should not fail to visit the Academedy of Medicine, 17 West 43d Street. They will be welcome. If they can attend one of the section meetings, so much the better. The building itself is ideal, especially constructed for the needs and purposes of the Academy and containing everything that can make the place useful and attractive for the members. Visitors will be most interested in the library, which, by the way, is the largest medical library in this country, excepting that of the Surgeon-General in Washington. There are upwards of 40,000 bound volumes and about as many pamphlets, and almost every day adds to the number. The Academy subscribes to every medical journal published, and when volumes are completed they are bound for permanent keeping. It thus becomes an easy matter for one to consult all the medical literature upon any subject, and the writing of a scientific paper is so much facilitated as to become almost a thing of joy.

The patient mentioned in my last letter with malignant disease (epithelioma) of the larynx, upon whom the preliminary tracheotomy was performed, has had his larynx extirpated After the tracheotomy it was decided to try the effect of iodide of potash, on the possible ground that the disease was syphilitic. This was discontinued after two weeks' trial, extirpation was decided upon, and all of the larynx, from the base of the epiglottis to the first ring of the trachea, was removed. It is in recent years that surgical skill has robbed malignant disease of the larynx of some of its terrors and dangers. Old methods of removing the larynx were usually followed by about forty per cent. of deaths, many of which were due to a secondary pneumonia, caused by wound secretions trickling down into the lungs. A method of operating, first devised by Bardeleben, I think, and first used in this country by Dr. Solis-Cohen in the remarkable case of Mr. Hickey, obviates this element of danger, and was the one employed in the above case. It consists essentially of shutting off the trachea from the wound while the latter is in process of healing. After the upper portion of the larynx has been removed the remaining trachea is dissected up for a short distance, separated from the esophagus, and brought forward above the sternum. The wound in the neck is closed behind the trachea, and the latter is protected from the oozings from the former by proper dressings. Such, very briefly outlined, was the operation as performed by Dr. Bodine in this case. The patient has made a good recovery thus far, he now breathes without embarrassment, and his general condition is much improved. This method of attacking malignant disease of the larynx is believed to mark a distinct advance in the surgery of this region. Although it is comparatively new, and has not been done a great number of times as yet, it has had sufficient trials to demonstrate its value. I should have mentioned that the above patient is already making encouraging progress in regaining his voice. By the way, the *New York Medical Journal* (September 5th) contains a good article by Dr. D. B. Delavan, Professor of Laryngology in the New York Polyclinic, which reviews the entire subject of recent advances in the surgical treatment of malignant disease of the larynx.

At a late meeting of the New York Academy of Medicine, Section on Rhinology and Laryngology, Dr. C. E. Munger discussed briefly, some "New Remedies in the Treatment of Diseases of the Upper Air Passages." He called attention to the use of acetanilid in the nose after operations. Application of the pure powder seems to favor rapid healing. He recommended formalin, one or two per cent. solution, as a deodorizer and disinfectant in purulent and syphilitic conditions of the nose and naso-pharynx. Pyrozone is valuable as an antiseptic and deodorizer. Two solutions had been employed, a four per cent. and a twenty-five per cent. He had used the latter with fair results in follicular pharyngitis and tonsillitis. For atrophic and ulcerative rhinitis he had used considerably ortho-mono-chloro-phenol. It lessens the crusts and secretions very promptly. He used a twenty-five per cent. solution in glycerine. The drug was especially useful in atrophic rhinitis.

Dr. C. C. Rice read a paper upon the "Methods of Controlling Nasal Hemorrhage." This hemorrhage, he said, was usually either from the septum or floor of the nose. He had seen only one case from the outer wall. If the hemorrhage were due to constitutional causes, renal or cardiac, etc., these of course should be treated. A general practitioner might resort to post-nasal plugging, but an expert, with a speculum and head mirror, should locate the bleeding point and insert a tight antiseptic tampon. The nostril should be plugged from the floor upward and forward. The bleeding point might also be touched with the galvano-cautery. Dr. F. J. Quinlan said that in these cases chromic acid, fused on a probe, was an excellent hemostatic.

Dr. W. F. Chappell made some remarks upon the *oleostearate of zinc* as a base for the application of medicines to the nose and pharynx. It is a semi-fluid substance, which does not absorb moisture, and which may be applied alone or incorporated with other agents.

Dr. B. Douglass presented a case of primary carcinoma of the inferior turbinated bone, a somewhat rare lesion of this part.

Dr. Holbrook Curtis described the case of a patient with so-called Rose Influenza, who always experienced a severe attack of this trouble whenever she passed a flower-stand. He had treated her with hypodermic injections of the extracts of the rose, lily of the valley, violet, heliotrope, and other choice varieties of perfumery, and after a year's treatment his patient could tolerate the presence of all sorts of flowers. Taking the "cue" from this case Dr. Curtis is now making some experiments upon hay-fever. He didn't say so, but I suppose these consist of the hypodermic injections of sweetly scented extracts of rye-grass, millet, rag-weed, blue-grass, clover, chenopodium, polygonum, etc., as the peculiar idiosyncrasies of each patient may require. Hay fever sufferers will doubtless await with interest the outcome of these novel studies in grass-extracts and therapeutics.

Dr. Rawlings, of Sandersville; Dr. Carson, of Griffin, and Dr. Brooks, of Atlanta, have been attending the Polyclinic.

L. B. GRANDY, M.D.

---

### RESUSCITATION FROM CHLOROFORM.

Resuscitation from chloroform is effected by the König-Maas method as follows: The operator, standing on the left side of the patient and facing him, places the ball of the thumb of the opened right hand upon the patient's chest, at a point between the apex beat and the sternum. He then repeatedly presses in the thoracic wall with a quick, strong movement, at the rate of thirty to one hundred and twenty times to the minute. The efficacy of the method lies in its direct action on the heart, restoring not the respiration only, but the circulation also. If on a fresh cadaver the pericardium be quickly and forcibly compressed, it is easy to detect a distinct pulse wave in the carotid arteries; and the pupils will be found to contract as the blood fills the capillaries of the iris.—*Sanitary Era.*

